# Gut-heart axis disruption and LPS translocation: driving atrial fibrillation through inflammatory storm and fibrotic mechanisms

**DOI:** 10.3389/fendo.2026.1787393

**Published:** 2026-02-25

**Authors:** Chunxiao Wang, Zili Xu, Peishuai Wang, Qianyu Zhang, Hongjie Xiang

**Affiliations:** 1 College of Traditional Chinese Medicine, Shandong Second Medical University, Weifang, China; 2 Shandong University of Traditional Chinese Medicine, Jinan, China; 3 Laoling People’s Hospital, Dezhou, China; 4 Department of Traditional Chinese Medicine, The First Affiliated Hospital of Shandong First Medical University & Shandong Provincial Qianfoshan Hospital, Jinan, China

**Keywords:** atrial fibrillation, gut microbiota, inflammation, lipopolysaccharide, oxidative stress, traditional Chinese medicine

## Abstract

Atrial fibrillation (AF) is one of the most prevalent arrhythmias in clinical practice, posing a significant threat to human health. The gut microbiota and its metabolites exert crucial effects on cardiovascular diseases via the gut-heart axis. Lipopolysaccharide (LPS), a component of Gram-negative bacterial cell walls, can enter the bloodstream when intestinal barrier function is compromised, triggering systemic inflammatory responses. Recent studies indicate that elevated LPS levels may increase the risk of AF onset through mechanisms such as promoting inflammation, oxidative stress, and myocardial fibrosis, and are associated with AF recurrence and poor prognosis. This review examines the role and mechanisms of LPS in the development and progression of AF, and explores potential strategies for preventing and treating AF by reducing LPS levels through approaches including gut microbiota modulation, anti-inflammatory diets, targeted inhibitors, and traditional Chinese medicine therapies.

## Graphical abstract

**Figure:**
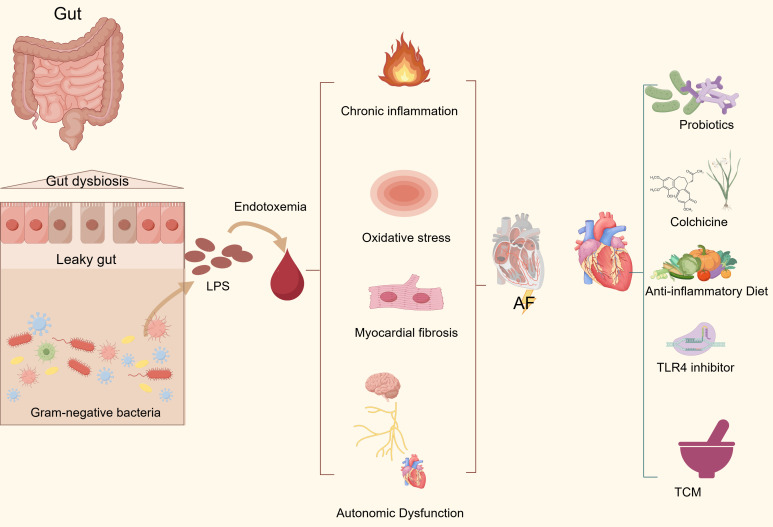

## 1 Introduction

Atrial fibrillation (AF) is one of the most common cardiac arrhythmias, characterized by disorganized atrial electrical activity and a pronounced decline in mechanical contractile function. These disturbances predispose individuals to left atrial thrombus formation, significantly increasing the risk of serious complications such as stroke, systemic embolism, and heart failure (1). Driven by an aging population and the increasing prevalence of cardiovascular risk factors, the global burden of AF continues to grow. Consequently, in-depth exploration of its pathogenesis and the identification of novel therapeutic targets have become critical priorities in cardiovascular research.

In recent years, with advancing insights into host-microbiome interactions, the gut microbiota—increasingly recognized as a “hidden metabolic organ”—has garnered significant attention for its role in cardiovascular health and disease (2, 3). Dysbiosis of the gut microbiota and imbalances in its metabolic products are hypothesized to contribute to the pathogenesis of various cardiovascular conditions through the gut-heart axis (4, 5). Lipopolysaccharide (LPS), a major component of the Gram-negative bacterial cell wall, is a potent endotoxin (6). Under normal conditions, an intact intestinal barrier confines LPS within the gut lumen; however, increased intestinal permeability allows LPS to translocate into the systemic circulation, inducing a state of chronic low-grade inflammation (7). A growing body of clinical and experimental evidence indicates that elevated circulating LPS levels are closely associated with the initiation, progression, and recurrence of AF (8). Chronic inflammation is a well-established key driver in the pathogenesis and sustenance of AF (9), and LPS-driven immune activation is believed to play a pivotal role in this process (10).

Nevertheless, the precise molecular mechanisms through which LPS promotes atrial remodeling—encompassing inflammation, oxidative stress, myocardial fibrosis, and autonomic nervous system dysregulation—are not fully elucidated. Therefore, this review aims to systematically synthesize recent advances in understanding the link between LPS and AF. It will critically examine the evidence across multiple pathophysiological levels, from endotoxemia and immune-inflammatory activation to distinct forms of atrial remodeling. The insights gathered herein are expected to provide novel theoretical foundations and potential intervention strategies for the early risk assessment, stratified management, and targeted therapy of AF.

## 2 Changes in plasma LPS levels in AF patients

Patients with AF frequently exhibit gut microbiota dysbiosis and compromised intestinal barrier function. This pathophysiological link is underscored by clinical observations, such as the higher prevalence of arrhythmias in septic patients compared to non-septic individuals (28% vs. 17%); notably, new-onset AF is the most common form, accounting for 67.8% of all arrhythmias in sepsis (11). Supporting the role of gut-derived endotoxins, a prospective clinical study revealed that AF patients not only had higher circulating LPS levels than those in sinus rhythm, but also that LPS may contribute to adverse cardiovascular events by enhancing platelet activation (12). Furthermore, Wang et al. demonstrated that plasma LPS levels following radiofrequency ablation can predict AF recurrence within one year (13). Consistently, elevated LPS levels during hospitalization have been significantly associated with non-valvular AF (13). Xu et al. found that the risk of AF in lung cancer surgery was associated with elevated levels of LPS (14). Collectively, these findings suggest that dynamic changes in LPS levels are closely associated with the development and progression of AF.

The aforementioned clinical studies consistently demonstrate that circulating LPS levels are significantly elevated in patients with AF, and its dynamic changes are closely associated with the occurrence, progression, recurrence, and prognosis of AF. These findings provide strong clinical correlative evidence for the role of the “gut-heart axis” in the pathophysiology of AF. However, when interpreting these observational data, it is essential to carefully consider their inherent limitations.

Current research primarily reveals a significant correlation between LPS and AF but cannot fully establish the direction of causality. A key and unresolved question is: Is elevated LPS a initiating factor in AF, or is it a consequence of AF (especially when accompanied by heart failure), which leads to systemic inflammation, hemodynamic disturbances, and secondary intestinal congestion and barrier dysfunction? Furthermore, common AF comorbidities such as aging (15), obesity (16), and diabetes (17) can themselves cause gut microbiota dysbiosis and barrier dysfunction, thereby inducing LPS translocation. AF patients often present with multiple comorbidities, such as heart failure (18), hypertension (19), diabetes (20), and chronic kidney disease (21), and are frequently on long-term medications, including antibiotics, proton pump inhibitors, and antiarrhythmic drugs. These comorbidities and medications themselves are known to significantly alter the structure and function of the gut flora (22, 23). Consequently, it remains difficult to definitively determine whether the “AF-associated microbial signatures” observed in studies are a cause, a consequence, or an epiphenomenon of AF. Furthermore, diet is the strongest environmental factor shaping the microbiota. Variations in geographical regions, cultural backgrounds, and individual dietary habits can lead to difficulties in replicating and generalizing study findings (24). Host genetic background may also simultaneously influence susceptibility to AF and the colonization of the gut flora (25), further adding to the complexity of the analysis.

To address these complexities, future research should employ more refined and diverse study designs to clarify causality. Prospective cohort studies can help establish temporal sequence by examining whether baseline LPS levels predict new-onset AF. Interventional trials (e.g., using probiotics, gut barrier protectants, or LPS-targeting inhibitors) can directly test whether modulating the gut–heart axis alters AF outcomes, providing the most direct causal evidence. Furthermore, leveraging genetic tools such as Mendelian randomization analysis can, to some extent, control for confounding factors, offering supplementary evidence for a potential causal relationship between LPS and AF. In study design, it is essential to systematically collect and adjust for key covariates, including comorbidities, medication use, diet (e.g., using standardized dietary assessment tools), and genetic background, to enhance the reliability and generalizability of the findings.

## 3 Risk factors for LPS and AF

### 3.1 Chronic inflammation

Inflammation, which constitutes the host’s immune response to injury or infection, plays a pivotal role in the development of AF. Chronic inflammation, in particular, serves as a fundamental mechanistic bridge linking LPS to AF pathogenesis (26). When LPS enters the circulation due to intestinal barrier dysfunction or other pathways, it binds to pattern recognition receptors—primarily Toll-like receptor 4 (TLR4)—on the surface of immune cells (e.g., monocytes and macrophages) and cardiomyocytes (27, 28). The activation of TLR4 initiates the downstream NF-κB (Nuclear Factor kappa-B) signaling pathway, a key regulator of inflammation (29). Subsequent nuclear translocation of NF-κB leads to a significant increase in the production of inflammatory cytokines, including IL-6, IL-1β, and TNF-α (29). These mediators create a persistent inflammatory microenvironment that exerts multifaceted detrimental effects on the atrial myocardium. Supporting this, animal studies have confirmed that LPS administration elevates pro-inflammatory cytokines in the pericardium of rabbits and promotes atrial arrhythmias through a TLR4-dependent mechanism (30, 31). Correspondingly, clinical studies report that serum levels of LPS, TLR4, and the aforementioned inflammatory factors are significantly elevated in AF patients compared to those in sinus rhythm, with their concentrations showing a positive correlation with AF duration (32). Furthermore, LPS-related inflammation can induce atrial conduction heterogeneity, thereby increasing the likelihood of AF recurrence. In summary, chronic inflammation driven by the LPS-TLR4 axis creates a ‘fertile ground’ for the initiation and perpetuation of AF.

### 3.2 Myocardial fibrosis

Myocardial fibrosis represents a core feature of structural remodeling in AF and a key pathological basis for its progression from paroxysmal to persistent forms (33). LPS is a potent pro-fibrotic driver. Animal studies indicate that gut microbiota dysbiosis can partially promote AF by elevating circulating LPS levels, thereby inducing atrial fibrosis (34). The underlying mechanism may involve LPS-induced upregulation of the inflammasome protein NLRP3, which enhances atrial fibrosis and facilitates AF progression. This is supported by the observation that administration of LPS-RS (an LPS antagonist) significantly reduces both the incidence and duration of AF in rats. Furthermore, Wang et al. reported a positive correlation between circulating LPS levels and serum TGF-β1, a central mediator of myocardial fibrosis (35, 36). Activation of the TGF-β1/Smad signaling pathway strongly promotes the synthesis and secretion of collagen (predominantly types I and III) by myofibroblasts, while simultaneously inhibiting matrix metalloproteinase (MMP) activity (37). It also upregulates their tissue inhibitors (TIMPs), leading to an imbalance between extracellular matrix (ECM) synthesis and degradation and resulting in excessive collagen deposition (38). Myeloid differentiation protein 2 (MD2) has also been implicated in cardiac fibrosis (39). MD2 binds to the extracellular domain of TLR4, facilitating the recognition of LPS and the formation of an LPS–MD2–TLR4 complex. Upon LPS exposure, the TLR4/MD2 complex recruits Toll/IL-1 receptor (TIR) domain-containing adaptors such as myeloid differentiation primary response 88 (MyD88) (40, 41), which in turn activates downstream signaling cascades, including NF-κB and mitogen-activated protein kinase (MAPK) pathways (42). Thus, MD2 plays a significant role in LPS-induced AF.

### 3.3 Oxidative stress

Oxidative stress arises from an imbalance between the production of reactive oxygen species (ROS) and the body’s antioxidant defense capacity, resulting in oxidative damage to biomolecules. Substantial clinical evidence indicates that oxidative stress plays a pivotal role in both the initiation and progression of AF (43, 44). LPS serves as a potent stimulus that triggers significant oxidative stress through multiple pathways (45). This is supported by the finding of Menichelli et al., who demonstrated that circulating LPS directly induces a state of oxidative stress (46). Within the cardiovascular system, major sources of ROS include NADPH oxidase (NOX), mitochondria, xanthine oxidase, and uncoupled endothelial nitric oxide synthase (eNOS) (47, 48). LPS has been shown to promote ROS production via mitochondrial dysfunction (49) and through the activation of NADPH oxidase, leading to the generation of superoxide, hydrogen peroxide, and hydroxyl radicals (50). This excess ROS contributes to impaired mitochondrial function and abnormal calcium handling in cardiomyocytes (51), thereby predisposing to AF (52).

Mechanistic insights from animal models further underscore this link. A study employing transesophageal pacing in NOX2 transgenic mice revealed that increased NOX2 expression elevates susceptibility to induced AF (53). Correspondingly, clinical observations in patients with community-acquired pneumonia have shown a positive correlation between NOX2 expression and AF occurrence (51), with NOX2 activation itself being associated with endotoxin exposure (54). Furthermore, LPS may promote AF by inducing cardiac iron overload and iron-dependent oxidative stress (55). Supporting this notion, an animal study utilizing an LPS-induced endotoxemia rat model reported that LPS-mediated disturbances in iron homeostasis can trigger the onset of AF (56). Previous clinical studies have similarly demonstrated that sepsis significantly alters systemic iron homeostasis, with elevated serum iron levels being independently associated with increased 90-day mortality in septic patients (57, 58). In addition, mitochondrial alterations in cardiomyocytes induced by LPS were found to correspond morphologically to those observed in siderophages (iron-laden macrophages) (59). The underlying mechanism may involve the activation of ferroptosis and the TLR4/NF-κB/NLRP3 inflammasome signaling pathway, which collectively contribute to sustained inflammation and dysregulated atrial pathological remodeling (60). In summary, by triggering robust oxidative stress, LPS directly contributes to both electrical and structural remodeling in the atria, thereby serving as a critical link between systemic inflammation and atrial dysfunction in AF.

### 3.4 Autonomic nervous system dysregulation

An imbalance in the autonomic nervous system, comprising the sympathetic and parasympathetic branches, is a significant trigger for AF. LPS has been shown to modulate both vagal and sympathetic nerve activities. Research indicates that chronic low-level LPS infusion in rats activates TLR4 in the nodose ganglion (NG), where the cell bodies of vagal afferent neurons reside, leading to heightened vagal tone (61). Enhanced vagal activity releases substantial acetylcholine (ACh), which activates acetylcholine-sensitive potassium currents (I_k_ACh). This signaling cascade shortens the atrial effective refractory period, increases its spatial dispersion, and promotes rapid focal discharges from sites like the pulmonary veins, thereby precipitating AF (62–64).

Concurrently, LPS also potentiates sympathetic excitation. A study by Hao et al. reported that LPS exposure in pregnant rats increased renin-angiotensin system (RAS) activity and oxidative stress within the hypothalamic paraventricular nucleus (PVN), a key central regulator of sympathetic outflow, alongside the activation of renal sympathetic nerve activity (RSNA) (65). Chronic activation of the renin-angiotensin-aldosterone system (RAAS) can promote hypertension by increasing renin secretion, enhancing sodium reabsorption, and inducing renal vasoconstriction (66). Hypertensive heart disease, in turn, promotes myocardial hypertrophy and fibrosis, leading to left ventricular remodeling and creating a substrate for AF (67). In a subsequent intervention, Hao et al. demonstrated that co-administration of melatonin with LPS in pregnant rats attenuated RAS activity and oxidative stress in the offspring’s PVN, reduced RSNA and blood pressure, and increased urinary sodium excretion (68). These findings collectively underscore that LPS-induced inflammation and oxidative damage are key mechanisms driving sympathetic overactivation, renal dysfunction, and hypertension. In summary, LPS induces autonomic dysregulation by triggering excessive central and peripheral sympathetic activity, altering cardiac autonomic neural tone, and directly disrupting myocardial electrophysiology, thereby emerging as a significant proarrhythmic mechanism.

**Figure 1:**
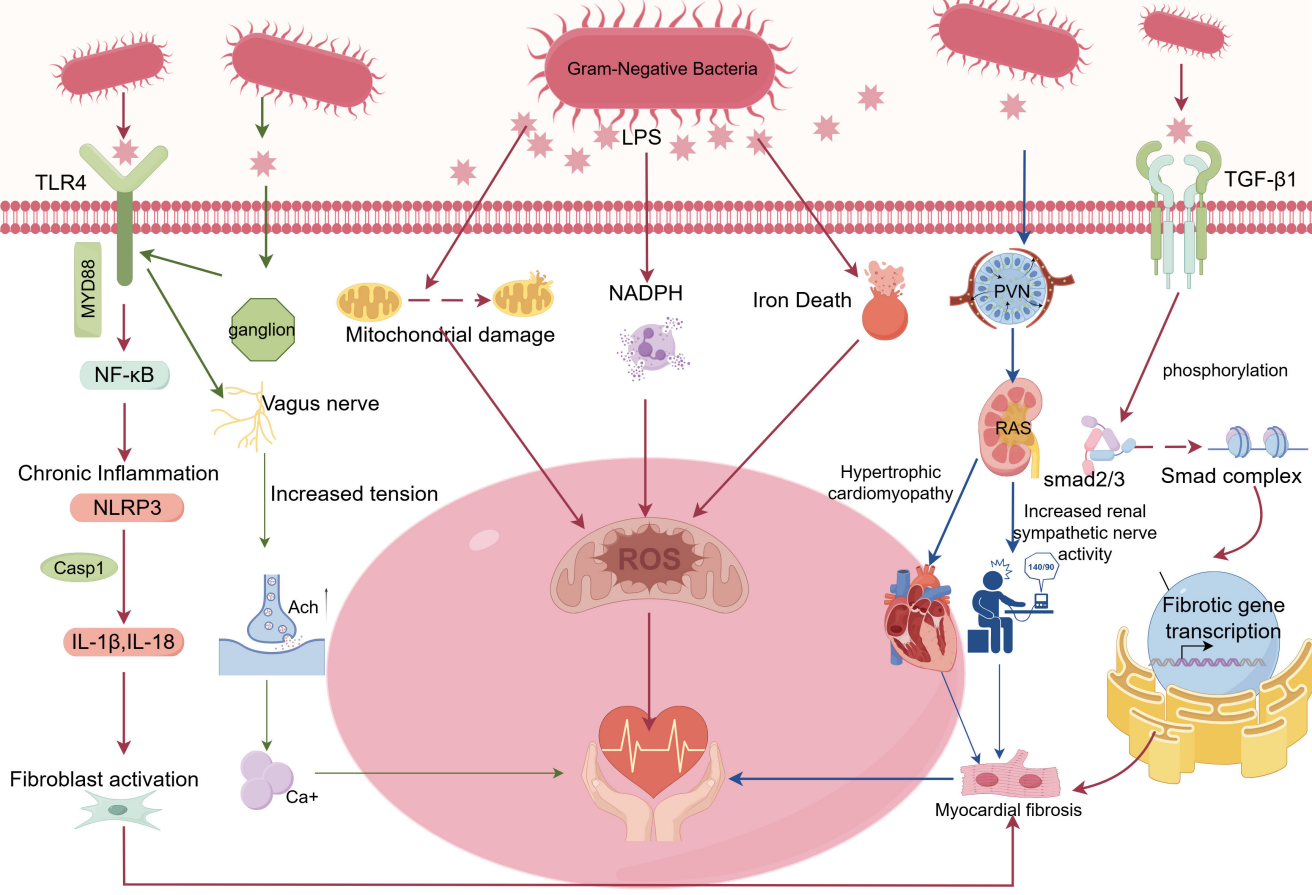
Mechanisms by which LPS induces AF.

LPS promotes AF not through an isolated pathway, but by establishing a complex pathophysiological network (**Figure 1**). This network is centered on chronic inflammation, supported by two core effector pillars—myocardial fibrosis and oxidative stress—and overlaid by autonomic dysregulation acting as a potent accelerator. These components are intricately intertwined and engage in causal interactions, forming a vicious cycle that collectively drives atrial electrical, structural, and autonomic remodeling, ultimately leading to the initiation and perpetuation of AF (**Table 1**).

**Table 1:** Analysis of the multi-mechanistic pathways underlying LPS-induced atrial fibrillation.

Mechanism classification	Key effect cells/tissues	Core signaling molecules/pathways	Principal pathological changes	Representative experimental/clinical evidence
Chronic inflammation	Immune cells (macrophages), cardiomyocytes	TLR4/MD2, NF-κB, NLRP3 inflammasome, IL-1β/IL-6/TNF-α	Inflammatory infiltration of the atria; shortening of the effective refractory period and increased dispersion in the atria	Animal model: LPS-induced pericarditis and atrial arrhythmia in rabbits; Clinical: Elevated serum LPS, TLR4, and inflammatory factors in AF patients, correlated with disease duration.
Oxidative stress	cardiomyocytes, endothelial cells	NADPH oxidase(NOX2)、Mitochondrial reactive oxygen species, Ferroptosis-associated proteins	Abnormal calcium handling in cardiomyocytes; mitochondrial damage; lipid peroxidation	Clinical: Expression of NOX2 in patients with community-acquired pneumonia is positively correlated with the occurrence of atrial fibrillation (39, 42); Animal studies: LPS induces atrial fibrillation in rats via the ferroptosis pathway (44).
Myocardial fibrosis	cardiac fibroblasts, myofibroblasts	TGF-β1/Smad, NLRP3 inflammasome	Excessive deposition of type I/III collagen; Rearrangement of the extracellular matrix	Research: Serum LPS levels correlate positively with TGF-β1 (23); Animal studies: LPS-RS (antagonist) reduces the incidence and duration of atrial fibrillation in rats.
Autonomic dysfunction	vagus ganglion, paraventricular nucleus of the hypothalamus, renal sympathetic nerves	Central RAS activation, acetylcholine currents, and sympathetic nervous activity (RSNA)	Increased vagal tone induces focal pulmonary venous discharges; excessive sympathetic activation promotes hypertension and structural remodeling.	Animals: Chronic LPS infusion activates TLR4 in the rat nucleus tractus solitarius (NTS), increasing vagal tone (49); LPS exposure in pregnant rats enhances the renin-angiotensin system (RAS) and oxidative stress in the paraventricular nucleus (PVN) region of offspring, alongside elevated renal sympathetic nerve activity (RSNA) (53).

### 3.5 Interactions and vicious cycles between mechanisms

The chronic inflammation triggered by the LPS/TLR4/NF-κB signaling axis serves as a central hub in the pathological network (69). It not only directly damages cardiomyocytes and the electrical conduction system (70), but also potently activates the transformation of cardiac fibroblasts into myofibroblasts by upregulating key factors such as TGF-β1 (71), promoting the excessive deposition of type I/III collagen, i.e., myocardial fibrosis (72). Conversely, the abnormal extracellular matrix (ECM) formed by fibrosis not only alters the anisotropy of electrical conduction but also provides a microenvironment for the retention and persistent activation of inflammatory cells, thereby amplifying and perpetuating the inflammatory response (73). Furthermore, inflammatory cells (such as activated macrophages) and cardiomyocytes damaged by inflammation or stress themselves become significant sources of ROS (74). Excessively produced ROS can further activate pro−inflammatory transcription factors like NF−κB (75, 76), forming a self−amplifying loop of “inflammation−oxidative stress.” Simultaneously, ROS directly attack cardiomyocyte membrane lipids, proteins, and mitochondrial DNA (77, 78), leading to cellular dysfunction and abnormal calcium handling (79), directly contributing to electrical instability. At the structural level, ROS can also activate matrix metalloproteinases (MMPs) (80), participating in the pathological remodeling of the ECM (81), thereby tightly coupling oxidative stress with the fibrotic process. LPS−induced autonomic nervous system imbalance is also deeply intertwined with this mechanism. On the one hand, enhanced vagal tone, through the release of acetylcholine, shortens the atrial effective refractory period and increases its dispersion, directly creating an electrophysiological substrate prone to reentry and triggered activity (82). On the other hand, LPS−induced sympathetic overactivation, mediated by the activation of central (e.g., hypothalamic paraventricular nucleus) and peripheral renin−angiotensin−aldosterone system (RAAS), not only elevates blood pressure and increases cardiac afterload (83), but also releases catecholamines (84) and angiotensin II (85)—which are themselves potent pro−inflammatory, pro−oxidative, and pro−fibrotic factors—thereby significantly exacerbating the processes of inflammation, oxidative stress, and fibrosis.

**Figure 2:**
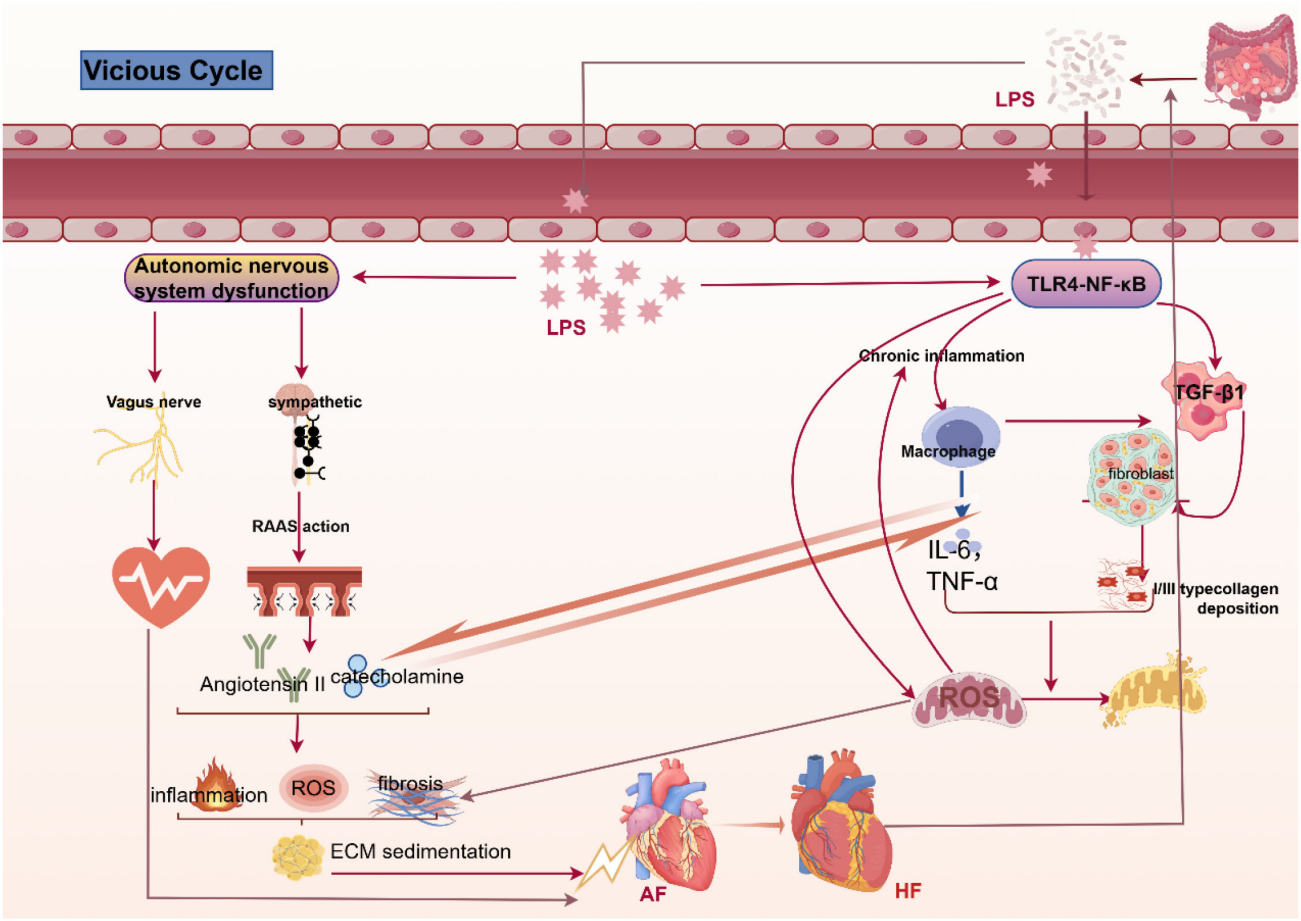
LPS drives the dynamic pathophysiological network of AF initiation and progression via the gut-heart axis.

Collectively, these mechanisms constitute a self-perpetuating vicious cycle (**Figure 2**): Intestinal barrier dysfunction leads to LPS translocation into the bloodstream, triggering systemic inflammation and oxidative stress. These processes directly damage atrial tissue and induce autonomic nervous system imbalance via sympathetic activation and RAAS. Inflammation, oxidative stress, and neuroendocrine activation together promote atrial fibrosis and electrical remodeling; ultimately leading to the initiation and perpetuation of AF. AF itself, particularly when it induces heart failure, causes intestinal congestion and hypoperfusion, which further impairs intestinal barrier function and increases LPS translocation. This perpetuates the entire cycle, relentlessly driving disease progression.

Therefore, understanding the mechanisms by LPS promotes AF requires shifting from a static “list of pathways” perspective to a dynamic “network interaction” viewpoint. Intervention at any single node may exert an inhibitory effect on the entire network, providing a theoretical foundation for multi-target therapeutic strategies targeting the gut-heart axis.

### 3.6 Lifestyle factors: take smoking as an example

Numerous studies have now confirmed that multiple lifestyle factors can increase the risk of developing AF by influencing the gut microbiota, such as high-fat diets (86), physical activity (87, 88), and aging (34). It is noteworthy that, in addition to traditional lifestyle factors, emerging evidence suggests smoking may also increase the risk of AF by disrupting the gut-heart axis. Smoking is a well-established independent risk factor for AF (89). Components in tobacco such as tar, nicotine, and carbon monoxide can increase heart rate and myocardial contractility, promote inflammation, cause endothelial dysfunction and thrombosis, reduce serum levels of high-density lipoprotein cholesterol (90–92), exert pro-inflammatory effects (93), and elevate the risk of developing atrial fibrillation (94). Recent research has revealed that it may participate in the development of AF through an indirect pathway by impairing the intestinal barrier and promoting LPS translocation. On one hand, smoking can cause direct damage to the intestinal microenvironment (95). Studies indicate that various harmful components in tobacco smoke can alter the permeability of the intestinal mucosal barrier, induce local oxidative stress in the gut, change the composition and thickness of the mucus layer, and disrupt the expression of tight junction proteins between intestinal epithelial cells, thereby significantly compromising the integrity of the intestinal epithelial barrier (96). This impairment of barrier function creates conditions for the translocation of lipopolysaccharide (LPS), a component of the cell wall of Gram-negative bacteria, from the gut into the portal vein and systemic circulation, potentially leading to or exacerbating “endotoxemia” (97). On the other hand, heavy metals contained in tobacco, such as cadmium, arsenic, chromium, mercury, and nickel, may be ingested and contribute to gut microbiota dysbiosis (98, 99). The LPS that enters the circulation can activate the TLR4/NF-κB signaling axis, triggering systemic chronic inflammation and oxidative stress. This, in turn, drives the initiation and progression of AF through multiple mechanisms, including promoting myocardial fibrosis, electrical remodeling, and autonomic nervous system dysfunction.

## 4 Treatment methods for AF associated with LPS

### 4.1 Probiotics

Probiotics are defined as live microorganisms that, when administered in adequate amounts, confer a health benefit on the host. They colonize the gastrointestinal mucosa and exert various clinical and immunomodulatory functions (100). Endotoxins such as LPS are primarily derived from Gram-negative bacteria, which often include putrefactive or transient flora. Supplementation with native probiotics—indigenous microorganisms that permanently colonize the gut—especially strains with immunomodulatory properties, can induce multiple beneficial biological effects (101, 102). Certain probiotic strains have been shown to influence the host and its immune system through diverse mechanisms. These include inhibiting the overgrowth of putrefactive bacteria by nutritional competition and niche exclusion, maintaining gut microbiota homeostasis, and enhancing the immune function of gut-associated lymphoid tissue. By activating relevant intracellular signaling pathways, probiotics can also suppress the synthesis and release of multiple inflammatory mediators, thereby further reducing endotoxin levels (103). Widely used probiotics for endotoxin clearance primarily include conventional strains such as lactic acid bacteria, streptococci, bifidobacteria, yeasts, and bacilli. For example, Lactobacillus plantarum, Lactobacillus delbrueckii subsp. lactis, and Bacillus licheniformis help modulate the gut microbiota, reduce LPS levels, and enhance intestinal epithelial barrier function (104). Wu et al. found that Lactobacillus rhamnosus activates the Wnt/β-catenin pathway, which stimulates the proliferation of intestinal epithelial cells, repairs epithelial damage, protects the intestinal mucosal barrier from inflammatory injury, reduces intestinal endotoxin leakage, and consequently suppresses elevated blood endotoxin levels (97).

Research indicates that specific probiotic strains, such as Lactobacillus reuteri and Lactobacillus rhamnosus, can ameliorate social behavioral deficits and alleviate anxiety in LPS-exposed rats (105). The underlying mechanism may involve probiotic cell wall components that mitigate LPS-induced systemic inflammation by modulating immune-inflammatory responses and oxidative stress (106). Furthermore, *in vitro* experiments have demonstrated that Lactobacillus reuteri and its metabolite gamma-aminobutyric acid (GABA) confer protection against acute ischemic cardiac injury induced by ischemia/reperfusion (I/R) surgery, primarily through the suppression of cardiac inflammation during the I/R process (107). Specifically, GABA inhibits lysosomal leakage and NLRP3 inflammasome activation, thereby blocking the polarization of macrophages toward the pro-inflammatory M1 phenotype and exerting anti-inflammatory effects. In summary, preventive oral administration of Lactobacillus reuteri effectively attenuates acute cardiac injury following I/R surgery by inhibiting myocardial inflammatory responses.

As a biological therapeutic agent, probiotics are widely used in clinical practice and functional foods. However, their live-microbial nature entails poor stability, susceptibility to environmental factors, and inconsistent efficacy across numerous clinical trials (108–110). Therefore, the adoption of probiotics as a routine treatment strategy requires further investigation.

### 4.2 Treatment with colchicine

Colchicine, an alkaloid derived from Colchicum autumnale, is a well-established treatment for gout and familial Mediterranean fever (111). Owing to its potent anti-inflammatory properties, it has been incorporated into clinical guidelines as a standard therapy for pericarditis (112). Given the central role of inflammation in the pathogenesis of numerous cardiovascular diseases, including atherosclerosis and AF (113), anti-inflammatory strategies have emerged as a promising therapeutic approach for these conditions (114). Colchicine, in particular, has attracted significant interest as a potential oral cardiovascular agent due to its low cost, straightforward extraction, favorable patient tolerability, and direct action on inflammatory pathways (115, 116).

As one of the most extensively studied NLRP3 inflammasome inhibitors, colchicine acts by inhibiting microtubule polymerization, thereby preventing the assembly and activation of the NLRP3 inflammasome (117). The NLRP3 inflammasome serves as a critical downstream hub for LPS signaling; its activation triggers caspase-1-mediated maturation and release of potent pro-inflammatory cytokines such as IL-1β and IL-18 (118). By inhibiting this pathway, colchicine effectively reduces inflammatory infiltration and fibrosis in atrial tissue. Animal studies have demonstrated that colchicine improves survival and left ventricular systolic function in mice following coronary artery ligation, concurrently reducing myocardial mRNA expression of NLRP3 inflammasome components (119).

Clinically, low-dose colchicine is associated with reduced long-term AF recurrence after ablation (120). Initiating colchicine from 7 days pre-ablation to 1 month post-procedure significantly diminishes the incidence of acute pericarditis and related hospitalizations, with higher survival rates observed in patients with paroxysmal AF receiving this treatment (121). Furthermore, multiple clinical trials consistently show that colchicine reduces the risk of ischemic cardiovascular events in patients with acute or chronic coronary artery disease (122–126). In summary, colchicine directly targets the downstream inflammatory cascade triggered by LPS, representing a key mechanism for controlling AF progression. However, its potential gastrointestinal side effects and risk of myelosuppression warrant careful clinical consideration.

### 4.3 Anti-inflammatory diet

The Western diet (WD) is characterized by high intake of monosaccharides, refined flour, salt, processed meats, animal fats, and food additives, coupled with low consumption of fiber, minerals, vitamins, and antioxidants (127, 128). This dietary pattern influences host metabolism and health by promoting weight gain, activating immune responses, and facilitating the development of several chronic metabolic diseases—including obesity, type 2 diabetes, cardiovascular disease, neurodegenerative disorders, and autoimmune conditions (129). Under the metabolic stress imposed by a WD, profound alterations in gut bacterial composition occur, which compromise intestinal barrier integrity and may subsequently lead to endotoxemia and systemic inflammation (130). It has been suggested that chronic high-fat diets create favorable conditions for the proliferation of gram-negative bacteria (131) and are also considered a cause of impaired intestinal barrier function (132). A high-fat diet is widely thought to impair the intestinal barrier, leading to elevated circulating levels of LPS and the development of a pro-inflammatory state.

In contrast, the Mediterranean diet (MD) is rich in monounsaturated and polyunsaturated fatty acids (MUFAs and PUFAs). In addition to these lipids, it also provides considerable quantities of fiber, antioxidant vitamins, and phytochemicals (133). Research has found that, significantly increases the proportion of Firmicutes (134), which can produce butyric acid with anti-inflammatory effects, and plays an important role in maintaining microbial homeostasis and reducing LPS production (135). Clinical evidence indicates that long-term adherence to MD is associated with significantly lower plasma LPS levels in the elderly compared to a WD (136). This reduction in endotoxemia is linked to MD’s beneficial effects on overall metabolic health, gut microbiota, and inflammatory status (137).

### 4.4 Targeted TLR4 inhibitors

Patients with cardiovascular disease, including those with AF, frequently exhibit intestinal barrier dysfunction and compromised integrity. This impairment allows LPS to translocate into the systemic circulation (138). Upon entry, LPS binds to TLR4 on immune cells and activates the release of pro-inflammatory cytokines, thereby initiating systemic inflammation. Specifically, via the TLR4/NF-κB pathway, LPS promotes the secretion of inflammatory mediators such as IL-1, IL-6, and TNF-α from macrophages and dendritic cells, which further exacerbates endotoxemia (139).

Given this cascade, directly blocking the interaction between LPS and its receptor represents the most upstream strategy for interrupting LPS signaling. TAK-242, a small-molecule inhibitor, selectively binds to the Cys747 residue in the intracellular domain of TLR4, thereby disrupting the interaction between TLR4 and its downstream adaptor proteins (TRAM/TRIF) (140). Animal studies have demonstrated that gut microbiota dysbiosis exacerbates cardiac dysfunction by activating the TLR4/NF-κB signaling pathway via LPS, which in turn drives myocardial inflammation and fibrosis (141). In this context, TAK-242 has been shown to significantly improve cardiac function and reduce fibrotic areas through a dual mechanism: inhibiting p65 phosphorylation and restoring microbial homeostasis. Another animal study confirmed that TAK-242 suppresses triggered activity induced by rapid pacing in both control and ischemia-related groups (30).

These findings not only enrich the theoretical framework of the gut-heart axis but also provide a novel rationale for targeting the intestinal microenvironment in the treatment of AF.

### 4.5 Traditional Chinese medicine

Traditional Chinese medicine (TCM) has a long history in China and, unlike some Western drugs known for adverse effects, exerts synergistic therapeutic actions through crosstalk between signaling pathways. Grounded in holistic philosophy and the theory that “the heart and small intestine are internally-externally connected,” TCM provides a unique perspective for the prevention and treatment of AF via multi-targeted, system-level regulation. According to TCM theory, pathological factors such as damp-heat and stagnation originating in the intestines—particularly the small intestine—may ascend to affect the heart, disturbing mental tranquility and thereby inducing palpitations. This view aligns closely with the modern concept of the “gut-heart axis.” In TCM, the pathogenesis of AF often involves heart meridian stasis and hyperactivity of heart fire. The systemic chronic inflammation and oxidative stress triggered by LPS entry into the bloodstream due to gut microbiota dysbiosis can be viewed as microscopic manifestations of TCM pathological factors like “stasis” and “fire”.

Both single herbs and TCM compound formulas can modulate LPS to reduce cardiovascular risk. Astragalus polysaccharides (APS), a macromolecular active component extracted from Astragalus membranaceus, exhibit multiple biological activities including immunomodulatory, antitumor, and antioxidant effects (142, 143). APS alleviates endothelial dysfunction by inhibiting LPS-induced macrophage polarization toward the M1 phenotype (144) and mitigating endothelial cell injury induced by H_2_O_2_, hyperglycemia, and TNF-α (145). Puerarin, derived from the root of Pueraria lobata, has been shown *in vitro* to significantly reverse the decline in H9C2 cell viability induced by LPS through inhibition of apoptosis (104). LPS markedly elevates ROS in H9C2 cells, while puerarin attenuates LPS-induced mitochondrial damage by regulating dynamin-related protein 1 (Drp1) and mitofusin-1 (MFN1). Moreover, puerarin and Torin1 significantly suppress the progression of sepsis by reversing LPS-induced inhibition of mitophagy in H9C2 cells via p62, LC3B, Pink1, and Parkin, thereby protecting cardiomyocytes (146). Zhuang et al. further demonstrated that puerarin activates GAS6 receptor agonists and targets the PGAM5–VDAC1 axis to regulate mitophagy, inhibiting LPS-induced necroptosis in cardiomyocytes and reversing mitochondrial pathway-related cardiac injury (147).

In addition, Xu et al. showed through *in vivo* and *in vitro* studies that ginseng effectively attenuates cardiac remodeling after myocardial infarction, with its protective effects closely associated with modulation of the SIRT1 signaling pathway (148). *In vivo* experiments indicated that ginseng improves cardiac function in a dose-dependent manner, reduces type I/III collagen deposition and IL-1β/IL-18 levels, and inhibits the NLRP3 inflammasome and TGFβ-1/Smads signaling pathways. *In vitro* experiments confirmed that ginseng similarly suppresses collagen production, inflammatory responses, and these key pathways in TGF-β1–induced cardiac fibroblasts and LPS-stimulated macrophages. The SIRT1-specific inhibitor EX-527 further verified SIRT1 as a critical upstream target of ginseng (148). Jingjie Fangfeng Decoction (JF), composed of Schizonepeta tenuifolia and Saposhnikovia divaricata, has been reported by Li et al. to significantly suppress LPS-induced inflammatory responses and oxidative stress in macrophages. Its key mechanism involves activation of the STAT3/p53/SLC7A11 signaling pathway, thereby inhibiting ferroptosis (149). The study also identified 5-O-methylquercetin, hesperidin, and luteolin as potential bioactive constituents of JF (149).

In summary, TCM demonstrates unique potential in modulating LPS-related mechanisms for the treatment of AF, guided by its holistic perspective and syndrome differentiation approach. TCM interventions act across multiple pathological stages. They work to reduce LPS production and intestinal translocation at the source, block midstream LPS signaling pathways, and alleviate downstream inflammatory and oxidative damage. This approach highlights the synergistic therapeutic advantage of multi-component, multi-target strategies.

Although numerous studies have revealed the substantial potential of single herbs and compound formulations in treating cardiovascular diseases via LPS modulation, the translation of TCM wisdom into outcomes that are comprehensible and broadly applicable within modern scientific frameworks still faces several core challenges. Foundational TCM theories, such as the concept that “the heart and small intestine are internally-externally related,” provide macro-level, holistic guidance for treating cardiac disorders from an intestinal perspective. However, a major bottleneck lies in the precise and systematic “translation” and cross-validation of TCM therapeutic methods—such as “clearing heat and drying dampness” or “promoting blood circulation and removing blood stasis”—with modern molecular pathways involved in “microbiota modulation, barrier repair, and LPS reduction.” Current evidence remains fragmented, lacking an integrated framework that bridges TCM principles, methods, formulas, and herbs with contemporary biomedical mechanisms. The prevailing reductionist research paradigm excels in deconstructing the effects of isolated components such as puerarin or astragalus polysaccharides. Yet, it falls short of capturing the essence of TCM compound formulas, which rely on the multi-target synergy among sovereign, minister, assistant, and messenger herbs. For instance, the anti-inflammatory effect of Jingjie Fangfeng decocation reflects its holistic efficacy; attributing this activity solely to a few identified constituents may not fully elucidate its therapeutic mechanism. Thus, developing a new research paradigm capable of integrating in-depth target analysis with holistic assessment of compound synergies represents an urgent and critical challenge in the field. Furthermore, the efficacy of TCM interventions stems from the combined actions of multiple constituents, making quality control far more complex than that of single-chemical drugs. Factors such as the geographical origin and processing methods of herbal materials directly influence the content and activity of key components—for example, the bioactivity of astragalus polysaccharides is affected by numerous variables. These issues pose significant challenges to the reproducible validation of research findings and the standardized production of TCM products.

In summary, as summarized in **Table 2**, intervention strategies for LPS-related AF exhibit a diverse landscape. Currently, colchicine can directly act upon the LPS-induced inflammatory cascade, representing a key mechanism potentially regulating the progression of AF (150). However, due to the drug’s potential gastrointestinal side effects (151) and risk of bone marrow suppression (152), its clinical utility must be carefully weighed. As foundational strategies for modulating the gut microenvironment and systemic inflammatory status, probiotics and anti-inflammatory diets offer benefits primarily focused on long-term risk management. However, they face challenges such as variable efficacy and patient compliance. In contrast, TLR4-targeting inhibitors and TCM represent promising research avenues. The former targets upstream precision blockade, while the latter emphasizes holistic multi-target regulation. Both demonstrate unique intervention potential, yet require further rigorously designed clinical studies to validate efficacy, safety, and to advance standardization before achieving widespread clinical application.

**Table 2:** Comparative intervention strategies for LPS-related AF.

Intervention strategy	Primary mechanism of action	Level of evidence	Potential/confirmed benefits	Limitations/Potential side effects	Development stage
Probiotics	Regulates gut microbiota composition, competitively inhibiting pathogenic bacteria; enhances intestinal epithelial barrier function; exerts systemic anti-inflammatory effects.	Clinical studies (high result heterogeneity)	May reduce circulating LPS levels, improve systemic low-grade inflammatory states, or contribute to lowering AF incidence risk.	High strain specificity with significant efficacy variations between formulations; efficacy influenced by host baseline microbiota, diet and other factors, exhibiting insufficient stability; long-term safety data remains incomplete.	As a dietary supplement or adjunctive therapy; requires further large-scale, strain-specific clinical trials to validate its precise efficacy in AF prevention and management.
Colchicine	Inhibits microtubule polymerization, thereby blocking NLRP3 inflammasome assembly and activation, acting as a potent upstream inflammatory inhibitor.	Clinical RCTs, included in cardiovascular disease diagnosis and treatment guidelines	Confirmed to reduce AF recurrence risk post-cardiac surgery and catheter ablation; lowers pericarditis incidence.	Common gastrointestinal reactions (diarrhea, nausea); rare but serious side effects including bone marrow suppression, myopathy and neuropathy.	Off-label use; already recommended in some clinical guidelines for AF prevention in specific scenarios (e.g., pericarditis, post-ablation). Requires low-dose administration with monitoring for adverse reactions.
Anti-inflammatory diet	Increasing dietary fiber and antioxidant intake promotes proliferation of beneficial bacteria (e.g., butyrate-producing bacteria); reduces pro-inflammatory microbiota and LPS production; improves metabolic health.	Observational studies and selected interventional studies	Long-term adherence reduces systemic inflammatory markers and circulating LPS burden, demonstrating clear cardiovascular protective effects and potentially lowering long-term AF risk.	Long-term adherence poses a major challenge; onset is slow, necessitating integration as a lifelong lifestyle intervention; efficacy varies considerably between individuals.	Primary/secondary prevention through fundamental lifestyle interventions; constitutes the cornerstone strategy for managing overall cardiovascular risk in AF.
Targeted TLR4 inhibitor	Selectively binds to the intracellular domain of TLR4, directly blocking LPS-TLR4 signaling to inhibit subsequent inflammatory cascade reactions at the earliest stage.	Primarily preclinical research	Demonstrated in animal models to improve cardiac function, mitigate myocardial inflammation and fibrosis, theoretically enabling fundamental intervention in LPS-driven AF pathophysiology.	Lack of clinical data in AF patients; potential interference with normal host immune defenses, introducing infection risks; drug specificity requires validation.	Clinical translation research phase; offers novel precision targets for AF treatment, though efficacy and safety require future clinical validation.
Traditional Chinese medicine	Multi-component, multi-target synergy: often concurrently exhibits anti-inflammatory, antioxidant, autophagy/ferroptosis regulation, and gut barrier improvement effects.	Primarily preclinical research, supplemented by clinical observations	Offers significant holistic regulatory advantages with substantial potential for symptom improvement and systemic condition modulation; certain components (e.g., astragalus polysaccharides, puerarin) exhibit cardioprotective effects in experimental studies.	Extremely complex mechanism of action, difficult to fully elucidate; significant challenges in standardizing quality control for herbal material, processing techniques, and formulations; insufficient modern pharmacological validation.	Distinctive therapies undergoing modern research; embody a “systemic medicine” intervention philosophy, yet necessitate rigorous clinical trials to align with contemporary medical standards.

## 5 Summary and outlook

LPS serves as a pivotal bridge linking gut microbiota dysbiosis to the pathogenesis of AF. Through multiple pathways—including chronic inflammation, oxidative stress, myocardial fibrosis, and autonomic dysfunction—it collectively promotes atrial electrical, structural, and autonomic remodeling, ultimately driving the initiation and perpetuation of AF. Given the central role of LPS in AF pathophysiology, several strategies have emerged as promising interventions. These include modulating the gut microbiota, adopting anti-inflammatory diets, using targeted inhibitors such as TLR4 antagonists, administering colchicine, and applying multi-component, multi-target approaches rooted in TCM. Such strategies aim to reduce circulating LPS levels and block its downstream signaling, offering novel perspectives for AF prevention and management.

However, research in this field still faces several challenges alongside significant opportunities. Most current evidence originates from animal models and observational clinical studies, and establishing a definitive causal relationship between LPS and AF requires more refined experimental designs—such as conditional gene knockout models and microbiota transplantation studies. There is also an urgent need to systematically integrate and “translate” the macro-level “gut–heart axis” theory into micro-level molecular mechanisms. For example, elucidating how TCM methodologies like “clearing heat and drying dampness” or “promoting blood circulation and removing blood stasis” correspond mechanistically to the modern concepts of “microbiota modulation–barrier repair–LPS reduction” is essential to achieving substantive progress in integrated AF therapy.

Moreover, for complex interventions such as probiotics and TCM compound formulations, new research paradigms that go beyond single-component analysis must be established. These should comprehensively evaluate their holistic and synergistic effects while addressing challenges related to quality control and standardization arising from their complex composition.
